# Supplementary material for: Urea-Assisted Synthesis of Mesoporous TiO2 Photocatalysts for the Efficient Removal of Clofibric Acid from Water
Source: Materials (Basel). 2021 Oct 13;14(20):6035. doi: 10.3390/ma14206035 (PMC8540116; doi:10.3390/ma14206035)
Supplement: Supplementary file 1 [file materials-14-06035-s001.zip › materials-1351130-supplementary.pdf]

Supplementary Materials

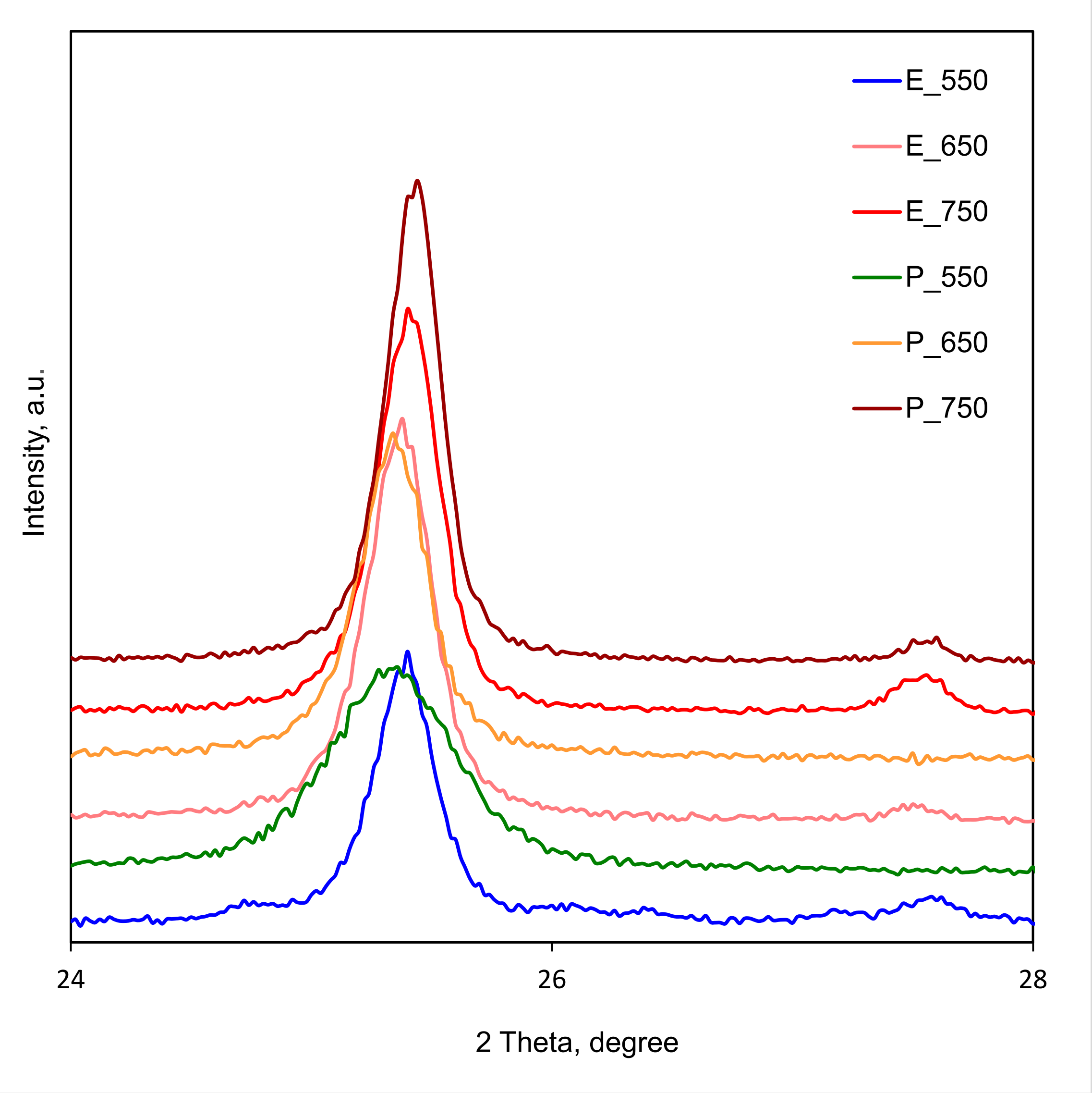

Figure S1. Details about the X-ray diffraction maxima width.

Sample **Magnification degree 10000 x** **Magnification degree 50000 x**

E-550

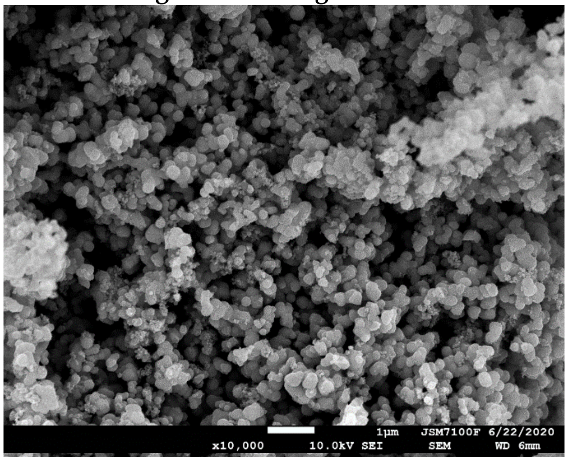

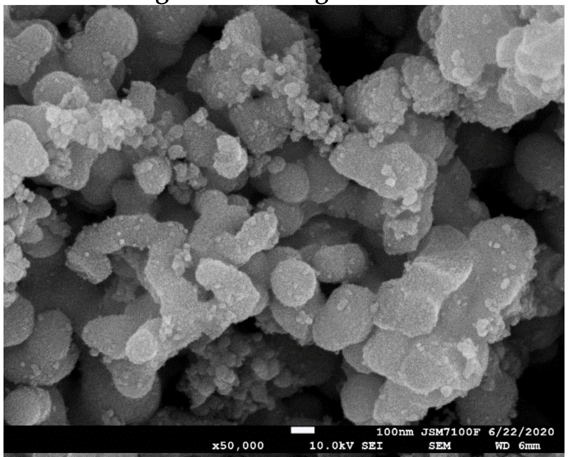

E-650

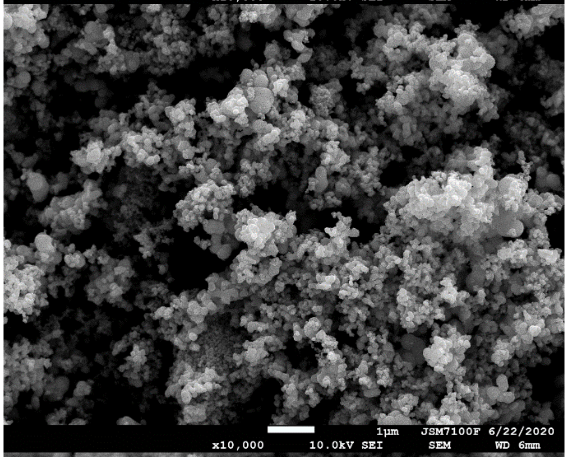

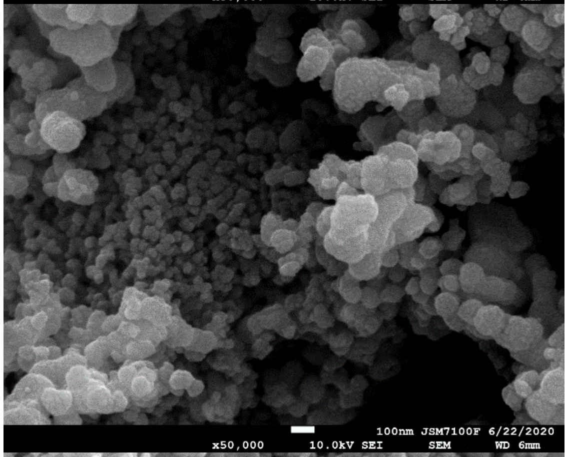

E-750

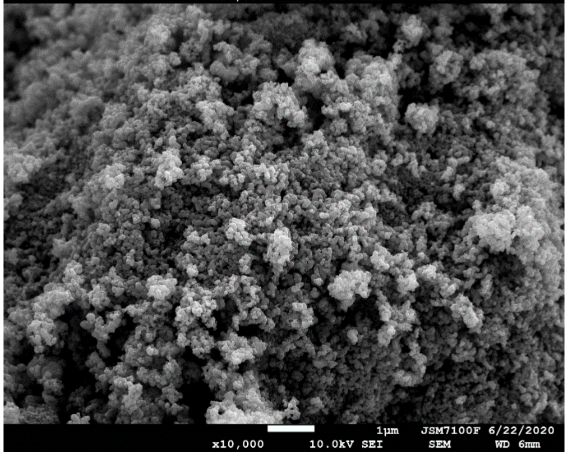

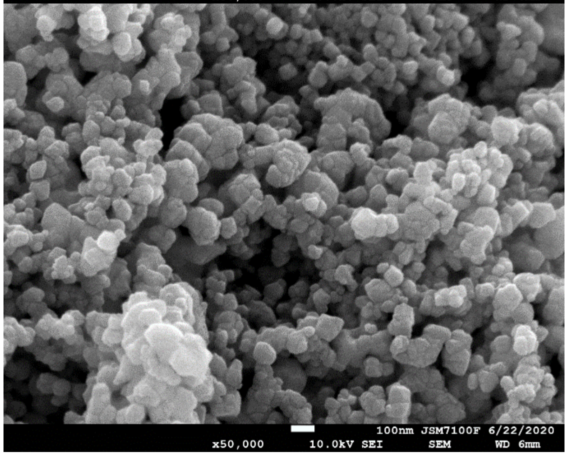

P-550

P-650

P-750

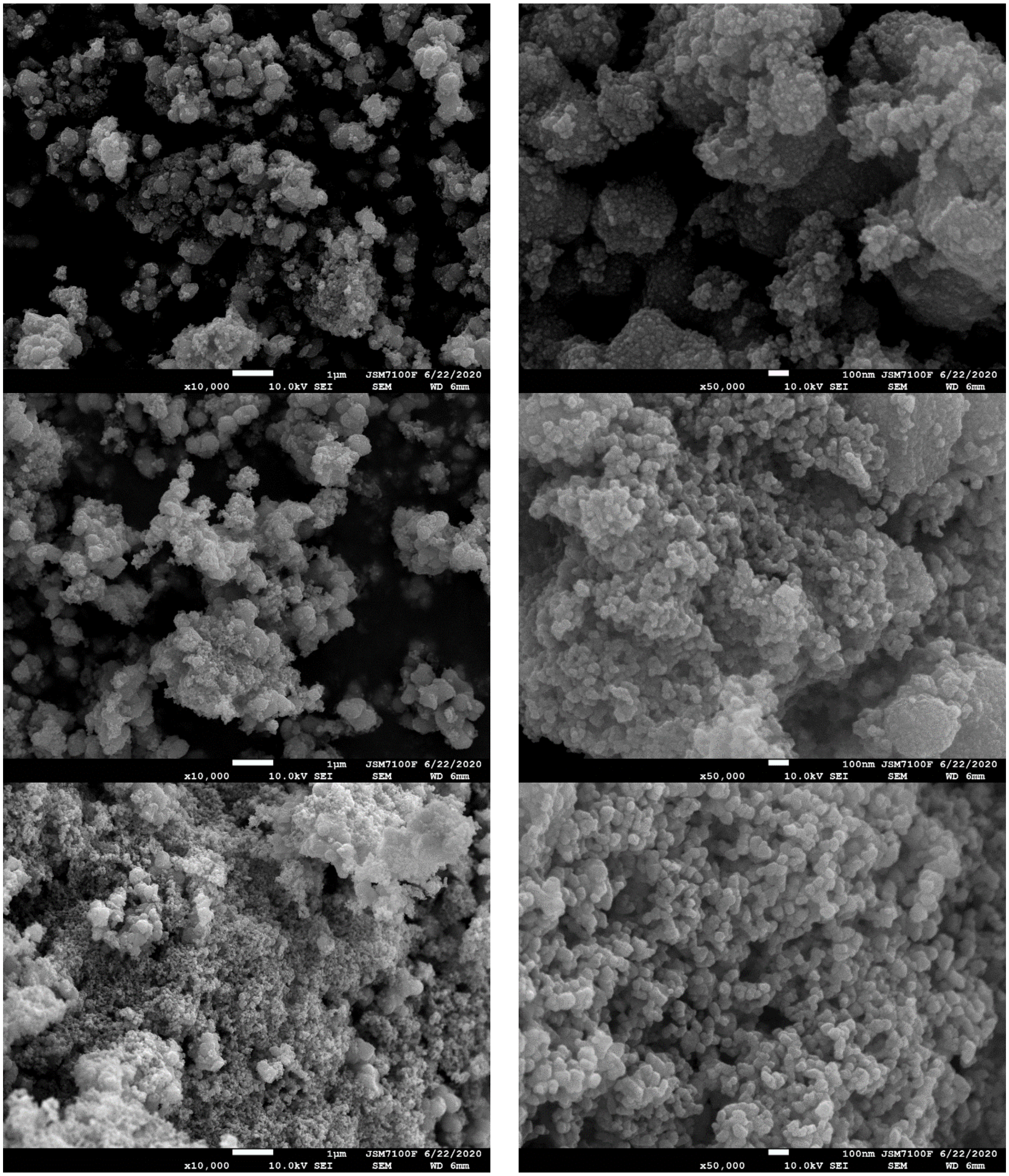

Figure S2. SEM images of the samples at different magnification degrees.
